# Observations on the Use of Arsenic as a Remedy for Tooth-Ache, or Destroying the Lining Membrane of a Tooth

**Published:** 1845-12

**Authors:** Amos Westcott


### ARTICLE III.

*Observations on the Use of Arsenic as a Remedy for Tooth-Ache, or for Destroying the Lining Membrance of a Tooth.* By AMOS WESTCOTT, M. D., D. D. S.

SINCE the alchemist abandoned his search after the grand catholicon, or universal remedy for all diseases, perhaps there has been no disideratum, involving consequences of no greater moment, than a cure for the tooth-ache, and some means of avoiding the universally dreaded operation of extracting these useful and ornamental organs. And it has ever been true, that as any achievement in the healing art has been deemed desirable, not only have efforts been correspondingly directed to it, but also have pretended remedies been offered to meet the exigency. Hence in every age, have such efforts been elicited, and remedies offered, to cure this justly styled "hell o' a' diseases." It would be greatly amusing to see collected, in one chapter, or rather one library, a history of all the ridiculous charms and nostrums, invented, and recommended, by the wise and the foolish, the learned and the ignorant, from the unlettered soothsaying monk, to the scientific surgeon, to overcome this evil.

This unconquerable dread of not only the tooth-ache, but of the extraction, and loss of the teeth, would seem, in many cases, paramount to every other; and hence the apprehension of imposition, by the most ignorant charlatan, is waived, provided there is the least hope that he can, with his charms or nostrums, soothe this maddening pain without extracting the tooth. We can thus only, account for sensible persons often entrusting their teeth to those, in whose judgment, or skill, they have not the slightest confidence, except as elicited by this delusive hope. A patient afflicted with tooth-ache, calls upon a respectable dentist for his advice and services.

On examination, the nerve is found to be exposed and inflamed, and the advice is to have the tooth extracted. The reply at once is "but cant you kill the nerve?" Perhaps the dentist in answer to this inquiry, informs him that although he can kill the nerve, this will by no means, be the end of the difficulty, and that he prefers not to serve him in this way.

Not unfrequently the result is, that the afflicted man declares, "I will not have my tooth out, that is settled, for Dr. —— says he can kill the nerve, and make the tooth as good as ever." The aid of the quack is, therefore, sought, and he "treats the tooth," and gets the fee.

Similar cases must come under the observation of every dentist in full practice, almost daily. When, therefore, we consider their frequency and their odious influence upon the profession, we can but feel that this subject deserves more special notice than it has hitherto received through the Journal.

In pursuing this subject, we shall notice some of the various ways, which have been adopted by those *having just claim to confidence*, to save teeth after the nerve is exposed—*especially* the present fashionable mode of *destroying nerves with arsenous acid.* The general object of each of them, excepting the practice of capping the nerve, is either to destroy its sensibility, or vitality.

Upon the subject of destroying the sensibility of exposed nerves, by the constant use of strong stimulants, such as the essential oils, kreosote, &c., no comment is needed, other than that they always prove prejudicial to the healthy state of the entire mouth.

No one ever attempted to retain such a tooth, by the use of such applications, without suffering infinitely more from the remote, if not from the direct effects, of this treatment, than was ever gained by their use. To say nothing of the effect of these substances themselves, either upon the mouth, or the general system, the fact that such teeth entirely preclude that cleanliness, absolutely necessary to the health and safety of the rest, is more than sufficient to condemn them. Another fact not unworthy of notice, is, that mastication is never performed on the adjacent teeth, often, and indeed generally, including all the teeth on that side of the mouth, both upper and under.

We have frequently seen the whole range involved in extensive disease, in this way—the teeth to the number of from ten to fourteen, encrusted with tartar, the gums spongy, and bleeding, the breath offensive, and all caused by a single exposed nerve kept quiet, by the constant application of some of the stimulants above named. In this way the foundation is not unfrequently laid, for the destruction of the entire set.

The habits of the American people, in respect to the preparation of their food, leave at best, far too little for the teeth to do, and if the exercise they generally get, is to be thus entirely lost on the affected side, the result is necessarily bad. In respect to *capping nerves* that are exposed, with the hope of saving the teeth by plugging them, it is a practice which so far as our own experience goes, by no means to be relied upon—nor do we think there is any good reason to hope, that it will ever prove satisfactory, when we consider the anatomy of the parts involved. A nerve cannot be exposed without a positive destruction of a portion of the lining membrane of the cavity, containing the pulp—nor is there any possibility of this being restored, unless we can believe that it will stretch across a foreign substance, which is impossible. It follows then that there is a wound inflicted, which cannot, on any supposition, be healed, and if not, we may expect trouble from it, sooner or later.

We have tried this experiment time, and again, without success in a single instance. And we are at a loss to account for the successful cases reported, unless we suppose a gradual destruction of the nerve, (which sometimes occurs without pain,) and this we consider to be the inevitable, ultimate result, if the plug is perfect.

Let us next examine the more prevalent practice of destroying nerves by the use of *arsenic*—a practice sanctioned by many of our best operators.

The use of arsenous acid, or the white arsenic of the shops, with the view simply to remove excessive sensibility of the bony structure of the teeth, has been very ably treated, and justly condemned, by Dr. S. Burr and Dr. Edward Taylor, in No. 2, Vol. 5, of the American Journal of Dental Science, although both of these gentlemen, either tacitly, or directly, sanction the practice, when the object is the destruction of the nerve.

Before giving any reasons for our own views upon this latter use of it, which are averse to its employment under any circumstances, we beg leave to say, that our prejudices have not arisen from any want of a faithful trial of its virtues, in perhaps some hundreds of cases. In expressing a belief that it would have been far better for the community and the profession, had not this remedy been discovered, or at least applied to this purpose, we do not expect to find ourself in the majority—we should certainly judge so, if we founded our opinion upon its almost universal employment. Our objections may be brought under the three following heads, viz.

1st. Its tendency to create a morbid action about the tooth.

2d. Its liability to produce bad, or even dangerous effects upon the general system, either through the agency of absorption by the vessels accompanying the nerve, or by its getting into the mouth and stomach, or by both.

3d. The prejudicial influence of this practice upon the profession.

In relation to the local effects of arsenic when applied to the nerve of a tooth, although its action is generally regarded, as simply escharotic, we think it by no means limited to this. We have in numerous cases removed the nerve, after the arsenic had been applied for twenty-four or thirty-six hours, and have uniformly found it highly injected, and to have taken on an entirely unnatural appearance, throughout its whole extent, although it may not have lost its sensitiveness. It is this rapid extension of this morbid action, which constitutes the basis of the first objection named. We hold, that if this morbid action extends *to*

the point of the root, there is no guarantee that it will not extend *through* it, so as to affect the parts exterior to the root. And such seems to be the result of experience; at least, so far as our own can furnish testimony upon the point. It may be remarked, however, that the tendency to periosteal inflammation, which this treatment induces, is considerably varied in the different classes of teeth. We have been in the habit of attributing this difference mainly to variation in the size of the nerves in the respective classes, and comparative amount of circulation and consequent freedom with which the effect was conveyed through the root. We have been led to this conclusion, not only from observing that this tendency to diseased action *about* the root, bore some ratio to the size of the nerve in the different classes referred to, but also from the fact that the inflammation and subsequent ulceration, in *molar* teeth, were generally confined to the palatine root, whose nerve is ordinarily many times larger than either of the others. That the nerves of the teeth or their accompanying vessels are capable of absorbing this substance, when of sufficient size, and especially when in a state of inflammation, there can be no manner of doubt, when we consider the abundant testimony of its frequently being absorbed, when used as a local application. We have tried it according to every recipe we have seen, and in all the combinations which have been invented, and all with a similar result. Although the morphine and kreosote, in the fashionable prescription, by their powerful narcotic effect, may diminish the pain, we cannot believe they prevent the result we have been describing—the absorption of the arsenic.

That inflammation about the roots of teeth, and alveolar abscesses, may result from, and often are induced by other causes, we of course admit. Indeed this is, as every dentist is aware, almost the uniform result, where the nerve dies by being long exposed to the common irritants of the mouth; yet this does not excuse us for employing as a remedy, an article whose use leads to a greater evil than that which it is designed to remedy. But beside the objection to this article, based on the danger of its being absorbed, and of thus creating an inflammatory tendency, and morbid action in the investing parts, we think the apprehension of danger from its effects upon the general system, is not entirely unfounded. No fact is more clearly established than that arsenic may be taken into the circulation by being absorbed when applied to a wound, and even upon the tongue. And when we consider, that a very small quantity, through this medium, has produced even fatal consequences, we feel that some more safe remedy is desirable, where the end to be gained is so trivial, compared with the danger of the means employed to effect it. We know it is, and will be claimed, that "in *judicious hands* no risk need be incurred." If the dentist did not place his prescription beyond his own power of control, this argument would have greater weight, so far as its action was concerned, by its getting into the mouth, and thence into the stomach; but placing it in the teeth of careless persons, he will frequently, on a second visit, look in vain, to find either the arsenic or its envelope, and ten chances to one, if his patient can give any account of it.

We may form some idea of the power of this remedy from the minuteness of the dose, when used in the treatment of disease, where health, or perhaps even life, is at stake. One of the most common forms of administration is that of Fowler's Solution. This consists of arsenic, four grains to one ounce of water, and four drops is considered a dose for an adult. This would make each dose contain one-thirtieth of a grain, and even this is directed to be given, and "the effect watched with the greatest caution, and to be discontinued on the first appearance of any untoward symptoms." Now, is not this many times less than is frequently placed in the cavity of a tooth even by "judicious hands," with no *certainty* of its not escaping into the mouth? In this view of the case, we have not considered the possibility of bad effects upon the general system, through the agency of absorption by the vessels of the nerve itself, and its being carried thus into the circulation, although we have good authority for believing, that "when applied *externally* to a wound, it occasions death, even more speedily than when it has been taken into the stomach."*

In our investigation of this subject, we have sought to ascer-

* Beck's Murry, p. 122.

tain the smallest fatal dose of arsenic when taken into the stomach, and also, whether its action as a poison, was peculiar, or what was its modus operandi. Upon either of these points, we have been unable to get definite information, although upon the former, we are enabled to arrive at an approximation to the truth. The action of narcotic poisons, and some of the irritant poisons, especially mercury, is varied so much by idiosyncrasy, that what would destroy life in the case of one, might prove perfectly harmless with respect to another. In regard to arsenic, although its action is doubtless varied to some extent, not only by peculiarity of constitution, but particularly by the state of health, yet we have no authority for supposing that the difference is ever so marked as in respect to the articles above referred to. The actual minimum dose, which would destroy life, is, therefore, not only rendered uncertain by such considerations, but still more, by the actual want of direct experiments upon this subject; it being, of course, inadmissible to experiment upon the living human subject. Although the cases of poison by arsenic are frequent, and are often investigated with the greatest care, yet when this article is given or taken, for criminal purposes, the dose is not only indefinite, but by no means the smallest, which may be supposed to prove fatal. The following testimony touching the question relative to the amount required to destroy life, in any case, and also, respecting its modus operandi, is from authors whose testimony should have great weight.

"Arsenic acts with great violence when taken internally, and destroys life in a very short time. When taken in *too large a dose*, as for instance, *one fourth of a grain*, it excites nausia, and slight chills. * * * *In doses above a grain, it destroys life*, with symptoms denoting inflammation of the alimentary canal." This idea (respecting its specific action) "is certainly countenanced both by the appearance on dissection, and by the corrosive quality of this substance."

"Mr. Brodie, however, rejects this explanation of its modus operandi, and thinks himself warranted to conclude, from several experiments he performed upon this subject, that arsenic enters into the circulation, and that it produces its effects by acting at once upon the *nervous system, the organs of the circulation, and the alimentary canal;* and that death depends immediately on the suspension of the functions of the brain, and heart." "This opinion is also entertained by Orfila."* Dr. George F. Gaeger of Stutgard, in a very elaborate and interesting dissertation, on the effect of arsenic upon various organized bodies, adduces a number of very strong arguments to prove, that arsenic does not produce its effects by any local action on the stomach and bowels. "Nor in the manner of acrid poisons, nor the nervous system, but is *analagous to the poison of the viper*, and ticunas, which *act primarily* upon the blood.†

It will be seen from the above quotations, that, upon the mode in which arsenic destroys life, there are various opinions, and by those having perhaps equal claim to authority.

The following testimony in respect to the minimum fatal dose, is somewhat more satisfactory, although not as definite as we could wish. Dr. Christison says, "the smallest actually fatal dose, I have hitherto seen *recorded*, is $4\frac{1}{2}$ grains." Dr. Pereira says, that, "the powerful effects sometimes produced, by $\frac{1}{8}$, $\frac{1}{4}$, or $\frac{1}{2}$ a grain, lead us to suspect that one or two grains might produce death, but we have no recorded case of this." Hahnemann says, "One, or two grains, may prove fatal," and Dr. Christison in remarking upon this statement, observes that "it cannot be very wide of the truth."‡ As arsenic is generally (at least when employed for dental purposes) used without weighing, it may be well to state in this connection, its great specific gravity, which is stated by Dr. Turner to be 3.7—nearly four times as heavy as the same bulk of water.

We submit the above suggestions and testimony upon this topic, hoping that they may elicit investigation and correct information upon this important subject. We would gladly leave this discussion, with our brief notice of the objections already stated, but we consider the third one named, hardly less formidable, than the two preceding. To the position that any article which it is *possible* to use safely, should be proscribed, either because it is liable to be

* Eberle's Therapeutics, p. 201.

† Edinburgh Med. and Surg. Journal, Jan'y, 1811.

‡ Pereira's Materia Medica and Therapeutics, vol. 1, p. 535.

abused by quacks, or because its use by men of respectability, may be calculated to increase the number, and enhance the power of empirics ; or because such practice tends to disgust the public with the resources of our art, we expect to find strong opposition. This, however, will not deter us from submitting our own views upon this subject, leaving it for the future to decide, whether they shall be set down as merely chimerical, or whether they should have a bearing in relation to this and other practices of similar tendency.

We shall be asked, why not strike arsenic, and a multitude of other articles, from the *materia medica?* This we think admits of a very brief answer. It has ever been regarded inadmissible, to use dangerous remedies, even where health and life are at stake, where the remedy is more dangerous than the disease, or when milder ones can be substituted. But the application of this article for *dental* purposes, has by no means the same apology. The object to be gained, at best, is merely the hope of saving a tooth. And were it true that even this object, could with certainty be realized, our objection would have less weight. But when we consider that *by its use*, we lay the foundation of morbid action about the tooth, resulting *generally* in an unhealthy abscess, an evil much more to be dreaded than the one for which the remedy was applied, we are inclined to say, that *far* "better that *one member* should *perish*," than incur the hazard of the experiment. But we will not *anticipate* objections to the sentiments we have expressed upon this subject, not doubting they will appear in due time.

Having taken the liberty to condemn the use of arsenic for the purpose of destroying nerves, we shall offer some remarks on what we consider a far preferable mode of practice, and our reasons for thus regarding it; we allude to the practice of removing them at once, with an instrument. A very excellent paper upon this mode of operating, was read by E. J. Dunning, before the American Society of Dental Surgeons, at its last annual meeting, and published in the first number of volume 6, of the American Journal and Library of Dental Science. Although we cannot by any means regard a tooth deprived of so much of its vitality, as it must be by the loss of its nerve, beyond contingency, yet from such experience as we have had, we consider the chances of success infinitely greater than when the nerve is destroyed with arsenic. In the one case, we make the operation strictly a surgical one, in the other we create a disease (certainly so far as regards the nerve itself) which we cannot control. When the nerve, in a healthy state, is removed with an instrument, it being cut off near its upper extremity, no morbid action is excited, nor any impediment formed to the healing of the wound, the absorbents being sufficient to dispose of any thing that need be left, between the upper extremity of the filling (which should be introduced as soon as hæmorrhage ceases) and the point of the root. This cannot be the case where the nerve is destroyed with arsenic, as then, there would be diseased action above the plug, certainly if the action of the arsenic has been such as to destroy the nerve its whole length. But if, in order to avoid this result, we only apply this substance so as to destroy the exposed portion, and then remove the remainder with an instrument, we have incurred the hazard of using this poison, to effect but a poor purpose, inasmuch as we only thus induce a *morbid* sensitiveness in the nerve, making the operation decidedly more tedious than if we had used the instrument without any previous treatment. We of course go on the supposition that the nerve is to be removed, "dead or alive," for no one would think of shutting in the nerve in either state, especially after it was saturated with arsenic. In respect to the pain necessarily connected with the removing of an inflamed nerve, it is seldom if ever very considerable. We have frequently performed the operation when the patient declared it no greater than would be inflicted by the prick of a pin.

But whatever may be said in favor of any method of destroying nerves, one objection must ever lie against the practice; which is, that it renders the teeth comparatively dead, and necessarily less capable of resisting morbid influences. Although we occasionally destroy the nerve of a tooth (exclusively by an operation) with the view to fill it, we cannot regard the indiscriminate practice with unqualified favor. The success of this practice must ever depend upon a strict regard to a variety of circumstances which materially modify the result. It is, for example, wholly inadmissible to destroy the nerve of a tooth in *any* way, which is already diseased about the root, and especially, if it has no antagonist. As a general rule, it may be said to be bad practice, where there is *general* disease of the gums, or an inflammatory tendency, and when the most rigid care is not taken by the patient, of the teeth and mouth. Not only should the state of the mouth be consulted, but temperament, the state of the health, and above all the habits of the subject are to be carefully considered, before we decide that the operation will, on the whole, be the least of two evils. Another governing circumstance, which should never be overlooked, is the class to which the tooth in question belongs. According to our own experience, it is much more successful upon the upper than upon the under teeth, although we are unable to assign a good reason for this. One thing is certain, that when inflammation and ulceration do result from this treatment, the consequences are far more serious, as connected with the under jaw, and the reason of this is obvious. The walls of the alveolar processes in the upper jaw are thin, and matter finds an easy egress, whereas, in the under, these walls are not only thick, but very dense and firm, making the escape of matter extremely difficult. Such is the resistance offered by the bony wall in these teeth, that it generally finds its escape by the side of the fang, often involving thus the entire periosteum. We have seen the inferior bicuspidati literally raised from their sockets in this way. Others may do as they please, but for ourself, we will not consent to destroy a nerve on the *under* jaw, in any manner, till our views are materially changed upon this subject, and we have not yet seen fit to extend this practice farther back than the first molares on the upper. Such are our own views upon what we consider the *best* method of destroying nerves. We have already given our objections to the practice of "treating" nerves with arsenic. It only remains for us to notice the basis of the extensive *call* for this practice, by patients, and the temptations to meet such calls by dentists. We have no doubt that a far greater proportion of the teeth which are treated, would be extracted, were it not for a supposed necessity on the part of the dentist that he must meet the *wish of his patient*, or there would be a risk of losing his patronage. In conversation a few days since with a professional brother, for whose honesty and skill we have much respect, and whose views upon the use of arsenic to destroy nerves, were similar to our own, he said that his "patient *would have* nerves killed," and that he "should lose one-half his business, did he refuse to do it." This, we have no doubt, is the feeling of many, yet we think it is making far too great a virtue, of a small necessity. With the present prevalence of the belief, that teeth may be saved in this cheap way, we will guarantee to any dental practitioner, that he will have an abundant call for this kind of service, yet can the call for such practice, unless it is in itself right, justify any one for adhering to it? Suppose that, by any means, the belief had become general, that arsenic would cure the head-ache, and it was by those affected with this malady, to be sought for this purpose, would the physician be justifiable for meeting the wish of his patient, knowing the prescription to be not only bad, but dangerous, simply because he feared he should lose his patronage?

Would a surgeon be justified in letting blood, against his own judgment, simply because he was requested to do so?

But there is not the slightest need of losing patients by refusing to kill nerves with arsenic. All that is necessary for a dentist to ensure confidence, is by his works, to convince the public in which he is located, that he is thoroughly qualified to serve them, and that he is willing to do so, in every way save by quack operations; let him inform his patients that when nerves are destroyed by *any "treatment," arsenic* is the material with which it is done, notwithstanding they may have been told, that it could be done with some "mild and harmless cordial;" let him above all, explain to his patient, the necessary, direct, and remote effects which this drug will have, and if all this is done in a kind, frank, and intelligent way, we should be willing to be responsible for the loss of every patient worth retaining. We claim that the call itself for this practice, is necessarily based on a false idea of its virtues, and ignorance of the danger and risk necessarily attending its application; and whenever patients are truly enlightened upon both, they, as a general rule, cannot be persuaded to have it applied.

No popular error connected with dentistry, is so universal, or so mischievous in its consequences as the supposition that a *tooth cannot ache after the nerve is destroyed.* This single error has been the basis of more quackery than all others combined. This mistaken notion, not only makes thousands willing dupes to those who will assure them that they have some peculiar method by which nerves may be destroyed, "without pain," but causes them ardently to seek the services of those who possess the invaluable specific. Its consequences are exceedingly bad in another respect. It serves as the basis of a negligence, both in respect to their own attention to their teeth, in regard to the use of the means of preserving them, and in respect to securing the timely services of the dentist; feeling that there is *one last hope*, "the nerve can be killed," and then all will be well. Any dentist who occupies the same field for any considerable time, is responsible in some measure, for the notions entertained by his patients, at least, upon all the important topics connected with his professional practice. He may prepare them for imposition, or he may, by diffusing correct information, shield them against the most insiduous attacks of empirics. Let, for example, a dentist who is at all entitled to respect, take good care to instruct his patients, that caries depends upon some peculiar state of the stomach, and as a conclusion, that these causes are beyond their control, and that any means adopted to secure cleanliness, other than those directed to keep the *front teeth looking decently*, are a humbug, let him take good care to have them all impressed with the idea, nay, the firm belief that teeth can be saved just as well, or perhaps rather better, after the nerve is exposed, so that it may be destroyed, and that all this can be done by a very slight operation, one which causes not the least pain, that all dead roots in whatever state they may be in, should be retained, "to keep the jaw in place, and the face from falling in," and we have no doubt that such an one will find ample scope for kreosote and arsenic, if for nothing else, for the *antiseptic* powers of these choice remedies. He will call about him just that class of patients which his doctrines are calculated to create, and who will demand at his hands, the very remedies he has advocated.

On the other hand, let him spare no pains to instruct his patients *properly*, in relation to all those cardinal points, constituting the basis of good practice, teach them the *real* causes of decay, how they may be avoided, how counteracted, in short, how controlled ; let him impress the belief, that even the nerves of teeth were placed there for some good purpose, and that the teeth are far more safe while the nerve is in its normal condition, and that the active ingredient in all the "mild cordials" used for the purpose of destroying them, is *ratsbane*, which can never be used without danger ; that the tooth must at best be a dead tooth, originating not only inflammation, but ulceration, and the most tedious and uncontrolable kind of tooth-ache ; that, indeed, the very means employed to cure, lays the foundation for all this train of evils, and he may rest assured that a very different kind of patients will grace his operating rooms, and with as different a suit of requests. Such instructions will not only secure to him who gives them, and practices accordingly, a suit of patrons who will stand by him, but they will free him from a thousand perplexities and trials necessarily accompanying *any* compromising course.

